# Mitochondrial autoimmunity and MNRR1 in breast carcinogenesis

**DOI:** 10.1186/s12885-019-5575-7

**Published:** 2019-05-02

**Authors:** Siddhesh Aras, Marie-Claire Maroun, Yeohan Song, Sudeshna Bandyopadhyay, Azadeh Stark, Zeng-Quan Yang, Michael P. Long, Lawrence I. Grossman, Félix Fernández-Madrid

**Affiliations:** 1 Wayne State University School of Medicine, Center for Molecular Medicine and Genetics, 540 E. Canfield Ave, Detroit, MI 48201 USA; 2 Department of Internal Medicine, Wayne State University, Detroit, MI 48201 USA; 3 Division of Rheumatology, Department of Internal Medicine, Wayne State University, Detroit, MI 48201 USA; 4 Department of Pathology, Wayne State University, Detroit, MI 48201 USA; 5 Department of Pathology, Henry Ford Health System, Detroit, MI 48201 USA; 6 Department of Oncology and Karmanos Cancer Institute, Wayne State University, Detroit, MI 48201 USA; 7 Wayne State University, University Health Center, 4H, 4201 St. Antoine, Detroit, MI 48201 USA

**Keywords:** Breast cancer, Metastasis, Mitochondria, Nuclear encoded mitochondrial gene, Autoantibodies

## Abstract

**Background:**

Autoantibodies function as markers of tumorigenesis and have been proposed to enhance early detection of malignancies. We recently reported, using immunoscreening of a T7 complementary DNA (cDNA) library of breast cancer (BC) proteins with sera from patients with BC, the presence of autoantibodies targeting several mitochondrial DNA (mtDNA)-encoded subunits of the electron transport chain (ETC) in complexes I, IV, and V.

**Methods:**

In this study, we have characterized the role of Mitochondrial-Nuclear Retrograde Regulator 1 (MNRR1, also known as CHCHD2), identified on immunoscreening, in breast carcinogenesis. We assessed the protein as well as transcript levels of MNRR1 in BC tissues and in derived cell lines representing tumors of graded aggressiveness. Mitochondrial function was also assayed and correlated with the levels of MNRR1. We studied the invasiveness of BC derived cells and the effect of MNRR1 levels on expression of genes associated with cell proliferation and migration such as Rictor and PGC-1α. Finally, we manipulated levels of MNRR1 to assess its effect on mitochondria and on some properties linked to a metastatic phenotype.

**Results:**

We identified a nuclear DNA (nDNA)-encoded mitochondrial protein, MNRR1, that was significantly associated with the diagnosis of invasive ductal carcinoma (IDC) of the breast by autoantigen microarray analysis. In focusing on the mechanism of action of MNRR1 we found that its level was nearly twice as high in malignant versus benign breast tissue and up to 18 times as high in BC cell lines compared to MCF10A control cells, suggesting a relationship to aggressive potential. Furthermore, MNRR1 affected levels of multiple genes previously associated with cancer metastasis.

**Conclusions:**

MNRR1 regulates multiple genes that function in cell migration and cancer metastasis and is higher in cell lines derived from aggressive tumors. Since MNRR1 was identified as an autoantigen in breast carcinogenesis, the present data support our proposal that both mitochondrial autoimmunity and MNRR1 activity in particular are involved in breast carcinogenesis. Virtually all other nuclear encoded genes identified on immunoscreening of invasive BC harbor an MNRR1 binding site in their promoters, thereby placing MNRR1 upstream and potentially making it a novel marker for BC metastasis.

## Background

Breast cancer (BC) is a leading malignancy in the United States with a very high incidence of metastatic disease [1]. National efforts to institute standard screening measures using annual screening mammography have led to enhanced early detection of BC, including a large number of slow growing breast tumors with relatively good prognosis [2]. However, aggressive BC is still a cause of significant mortality among women, with over 40,000 BC-associated deaths reported in 2017 in the United States [3], and the early ability to differentiate indolent from aggressive BC is still lacking [4]. Multiple studies have demonstrated that the sera from patients with BC and other solid tumors consistently contain a variety of autologous cellular antigens or tumor-associated antigens [4]. A plethora of autoantibodies is known to be present in BC serum; we recently reported that anti-mitochondrial antibodies (AMAs) constitute a distinctive feature of the autoantibody profile of BC sera and proposed that autoimmunity participates in breast carcinogenesis [5]. We further reported that 6 of the 13 mitochondrial proteins comprising the ETC are BC autoantigens [6].

In this work, we report the autoreactivity of two clones identified from cDNA of BC proteins encoding a sequence identical to the mitochondrial regulator MNRR1. The identification of the nDNA-encoded bi-organellar protein MNRR1, which functions in both the mitochondria and the nucleus, as a BC autoantigen offered proof of principle that AMAs in BC sera are the expression of mitochondrial autoimmunity. When localized in the mitochondria, MNRR1 binds to and activates cytochrome *c* oxidase [7, 8] whereas in the nucleus it functions as a transcriptional activator for genes harboring an 8-base pair DNA core of a conserved 13-bp element that responds maximally at 4% experimental oxygen concentration, and therefore is referred to as the oxygen responsive element [8–10].

MNRR1 expression has previously been associated with survival prognosis in a number of cancer types including lung [11] and liver cancers [12]; consequently, we explored the possibility of a direct role for MNRR1 in BC. In this work we show that MNRR1 is a breast cancer autoantigen that directly participates in breast metastasis. The present data supports our hypothesis that mitochondrial autoimmunity as well as MNRR1 auto-reactivity are involved in breast carcinogenesis. We further propose that detection of autoantibodies against MNRR1 in the sera of BC patients but not in control non-cancer sera suggests that MNRR1, alone or in conjunction with a panel of other AMAs, can contribute to the early diagnosis of BC and potentially differentiate indolent from aggressive disease.

## Methods

### Human subjects

Sera were prospectively obtained from a cohort of 100 women > 40 years of age undergoing annual screening mammography at Henry Ford Health System (HFHS), who had biopsy-confirmed IDC and 100 women with biopsy-proven benign breast disease (BBD), as previously reported [6]. Each of these women was invited to donate 10 mL blood samples after signing an informed consent. The demographic characteristics of cases and controls have been reported [6, 13]. This study was approved by the HFHS and Wayne State University (WSU) Institutional Review Boards (IRBs) (WSU protocol #0603003557, Human Investigation Committee #038306A; HFHS IRB #3798).

### Construction of T7 phage library

A random primer cDNA library of T7 phages was assembled using directional cloning of cDNA from BC cell lines using the Orient Express cDNA library construction system (Novagen, Billerica, MA). Since commercially obtained libraries are usually constructed from RNA isolated from a single malignant tumor, we constructed a multi-human BC cell line cDNA library considering the known heterogeneity of BC [14]. The established cell lines used for library construction included MCF-7, SKBR, T47D, SUM44, SUM102, SUM149, and SUM159. The BC cell lines were a gift of Drs. Frederick Miller and Stephan Ethier, Karmanos Cancer Institute, Wayne State University. Total RNA from the BC cell lines was isolated with a RiboPure Kit (Ambion, Austin, TX) according to the manufacturer’s instructions. For construction of a T7 phage display library, poly(A)^+^ RNA was isolated using Straight A’s mRNA Isolation System (Novagen) and then the T7 Select® 10–3 Orient Express™ cDNA Cloning System. In brief, 4 μg Poly(A)^+^ RNA were reverse transcribed into double stranded cDNA. After flushing the DNA ends, ligation of the cDNA to directional EcoR I/Hind III linkers, digestion of the cDNA by EcoR I/Hind III, and cDNA size fractionation, the prepared cDNA was inserted into T7 select 10–3 vector. The phage display cDNA library was constructed by packaging in vitro followed by plate proliferation. Plaque assay and PCR were used to evaluate the library. The resulting phage library contained 4.5✕10^6^ independent clones as determined by plaque assays. The library was amplified once by plate lysate amplification, resulting in a phage titer of 4.3✕10^10^ pfu/mL. After elution with Phage Extraction Buffer (20 mM Tris-HCl (pH 8.0), 100 mM NaCl, 6 mM MgSO_4,_) the eluate was centrifuged and stored at − 80 °C. The insert sizes of individual clones of the complete library were analyzed by PCR with the forward primer 5′-GGAGCTGTCGTATTCCAGTC-3′ and the reverse primer 5′-AACCCCTCAAGACCCGTTTA-3′.

Immunoreactivity was used as the main criterion for biopanning of BC sera, which was performed according to the manufacturer’s instructions (Novagen).

### Biopanning

Both immunoscreening of a cDNA display library of BC proteins using sera from patients with BC and high throughput autoantigen microarray analyses of the antigens recognized by BC sera have been described [6, 13]. Potential BC autoantigens were immunoscreened with a pool of sera from women with IDC of the breast, from ductal carcinoma in situ (DCIS), and from healthy women with BBD. The sera from BC patients used for immunoscreening contained high-titer AMAs (≥1:320–1:640) by immunofluorescence on HepG2-cells. A/G agarose beads (Santa Cruz Biotechnology, Santa Cruz, CA), incubated overnight with 100 *μ*L of a 1:50 dilution of cloning sera at 4 °C, were used to react with a culture of *Escherichia coli* strain BLT5403, which was then lysed and centrifuged. Up to 5 additional rounds of biopanning were then performed on the resulting supernatant. This is required because the initial rounds of biopanning do not yield individual unique phages but a mixture of phages with different inserts. Thus, biopanning is repeated until lysed clones yield a unique phage indicated by a single band by PCR and this usually takes up to 5 rounds of biopanning and occasionally more.

### Autoantigen microarray

An autoantigen microarray was constructed in triplicate on fluorescent array surface technology glass slides using a Flexys robot (Genomic Systems) as reported [6, 13]. Each slide printed with a phage clone, which had a single band by PCR, was hybridized with an individual serum from the collection of patients with IDC of the breast, or with a non-cancer control serum that showed BBD at breast biopsy. Slides were then treated with a mouse monoclonal antibody against the non-variable T7 phage coat protein (Novagen). CY3-labeled anti-human secondary antibodies and C5-labeled anti-mouse antibodies were used to assess patient autoreactivity and to quantify phage concentration, as previously reported [6, 13].

### Immunohistochemistry

Pathology-proven benign and malignant breast tissue specimens obtained during surgery for reconstructive mammoplasty or from specimens of IDC of the breast, respectively, were incubated with a specific antibody targeting MNRR1 and stained for IHC. To evaluate specimens, phase contrast images were obtained using a Leitz Laborlux 12 microscope equipped with a SPOT digital Camera System (version 4.5). Blind data analysis was performed using ImageJ software (ImageJ 1.48v, https://imagej.nih.gov/ij/). Briefly, a random area was chosen on the image (300 × 240 pixels, size kept constant for all images analyzed) and the intensity of staining was measured after obtaining the histogram. Three distinct areas were analyzed per sample, and an average staining intensity was calculated for each slide. Antibody staining was performed on independent benign (*n* = 3) and IDC (*n* = 4) samples. Normal breast tissue was also stained as a negative control and the value obtained was approximately 30% lower than the average of benign samples. Statistical significance was calculated using a two-sided Wilcoxon rank-sum test for 9 benign intensity values and 12 IDC values obtained from ImageJ.

### Cell lines for MNRR1 biochemical analyses

MCF7 and MCF10A cells were a gift from Dr. Leonard Lipovich (Wayne State University). HEK293 cells with WT-MNRR1 and MNRR1-KO have been previously described [15]. MCF7, MDA-MB-468, and HEK293 cells were grown in modified DMEM with 10% fetal bovine serum and 1% penicillin-streptomycin. The MCF10A cells were grown in DMEM-F12 medium with 5% horse serum and 1% penicillin-streptomycin supplemented with 20 ng/mL EGF, 0.5 μg/mL hydrocortisone, and 10 μg/mL human insulin. MNRR1-KO in MCF7 cells was performed as follows: cells were transfected with a MNRR1 human CRISPR/Cas9 KO Plasmid (Santa Cruz Biotechnology Cat # sc-412127) with the following gRNA sequences. a) Sense: GATGGTCTCACCTGGCCGGA, b) Sense: ATTACCTACCGTTTGCAAGT, and c) Sense: CCCACCTGGTAAGTGATGTC. A homology directed repair plasmid (Santa Cruz Biotechnology Cat # sc-412127-HDR) harboring the puromycin resistance gene was co-transfected using Transfast transfection reagent (Promega) per the manufacturer’s instructions. Puromycin at 1 μg/mL was used to select resistant clones. The control cells (transfected with a control vector) along with the knockout cells were maintained in the selection antibiotic. Antibiotic resistant clones were tested for knockout using the MNRR1 antibody described below.

### Reagents

MNRR1 antibody (Cat # 19424–1-AP) was purchased from Proteintech (Rosemont, IL). Rictor (Cat# 2114), pS473 AKT (Cat# 4060), panAKT (Cat# 8596), AKT3 (Cat# 4059), PGC-1α (Cat# 2178), GAPDH (Cat# 8884), Actin (Cat# 12748), and Tubulin (Cat# 9099) antibodies were purchased from Cell Signaling Technology (Danvers, MA). All plasmids used have been previously described [7, 8].

### Oxygen consumption measurement

Intact cellular oxygen consumption was measured in cells with a Seahorse XF24^e^ Bioanalyzer according to the manufacturer’s instructions. Cells were plated at a concentration of 40,000/well a day prior to the assay. Oxygen consumption is shown relative to the control set at 100.

### Live staining

Cells were grown on coverslips and stained with Mitotracker Green (Thermo Fisher, Waltham, MA) using the manufacturer’s protocol. Briefly, cells were incubated with 250 nM Mitotracker Green in serum free medium without phenol red at 37 °C and 5% CO_2_ for 15 min. Excess stain was removed by one wash with medium and the coverslips were mounted on a slide. A 63x oil immersion objective was used to capture live fluorescent images with a Zeiss Imager M2 equipped with ApoTome and AxioCam HRM cameras. Zen 2 Pro Software was used to process images. Intensity was measured with ImageJ software. The fluorescent images were inverted, and intensity was calculated by obtaining a histogram. Single cells were selected, and different fields were monitored. For each image, a small area in the background was chosen as blank and subtracted from the intensity value obtained from the histogram.

### Cell migration and growth assay

MCF7 cells with MNRR1-WT and MNRR1-KO genotypes were analyzed for their cell migratory capacity using a Corning® BiocoatTM Matrigel® invasion chamber according to the manufacturer’s instructions. Data have been represented as migration of the MNRR1-KO cells relative to the MNRR1-WT cells set to 100%. Cell growth was measured using the Cell Titre96 One solution cell proliferation assay (Promega, Madison, WI) according to the manufacturer’s protocol.

### RNA sequencing

Total RNA from MNRR1-WT, MNRR1-KO, and MNRR1-KO expressing a transcriptionally active version of MNRR1 (MNRR1-TA) was isolated. Samples with a RIN ≥8 were used for further processing. Indexed (barcoded) libraries were generated using the Illumina TruSeq Stranded Total RNA Library preparation kit. Sequencing of the libraries was performed on the lllumina’s HiSeq 2500 next-generation sequencer. The sequencing read data was converted to a text-based FastQ format for analysis using Base Space Sequence Hub (Illumina, San Diego, CA).

### Immunoblotting

Protein levels were analyzed using specific antibodies as described previously [7, 8].

### Statistical analysis

The statistical significance of the reactivity of the MNRR1 clone on the autoantigen microarray was calculated individually as previously reported [6]. PCR, sequence analysis, hybridization of the printed phage on glass slides, and development of the auto-antigen microarray were also performed as previously reported [6]. The homology search of the sequence of phage 15, which revealed identity to an MNRR1 sequence, was performed using the Basic Local Alignment Search Tool (BLAST) and confirmed by BLAST-like Alignment Tool (BLAT) [16, 17].

Statistical analyses for the MNRR1 biochemical studies were performed using MSTAT version 6.1.1 (N. Drinkwater, University of Wisconsin, Madison, WI). Two-sided Wilcoxon rank sum tests were used to calculate the statistical significance.

## Results

Immunoscreening of a T7 cDNA display library of BC proteins constructed with messenger RNA from established BC cell lines and biopanned with BC sera rich in AMAs led to the cloning of a large number of expressed sequence tags (ESTs), 184 of them representing unique gene products with significant immunochemical reactivity with BC sera. Of these unique gene products, 87 phages had sequences encoded by mtDNA that were highly associated with the diagnosis of IDC [6]. In that work, 6 of the 13 mtDNA encoded proteins of the ETC were identified as BC autoantigens, including 67 clones of ND5 and one clone each of ND4, ND4L, ND6, ND6, COXI, MT-ATP6, and 16S rRNA (MTRNR2, encoding humanin; NC_012920.1; Q8IVG9.1) [18] and a panel (Table 1 and unpublished) of nDNA-encoded mitochondrial proteins recognized as autoantigens by BC sera but not by non-cancer control sera. Another one of these antigens, not included in Table 1, is MNRR1, the subject of this report.

**Table 1:** Mitochondrial DNA-encoded gene products recognized by the immune system as autoantigens

Antigen	Accession
ND4	NC_012920.1 YP_003024035.1
ND4L	NC_012920.1 YP_003024034.1
ND5	NC_012920.1 YP_003024036.1
ND6	NC_012920.1 YP_003024037.1
COXII	NC_012920.1
ATP6	NC_012920.1 YP_003024031.1

Mitochondrial genome encoded proteins identified as autoantigens

**Fig. 1:**
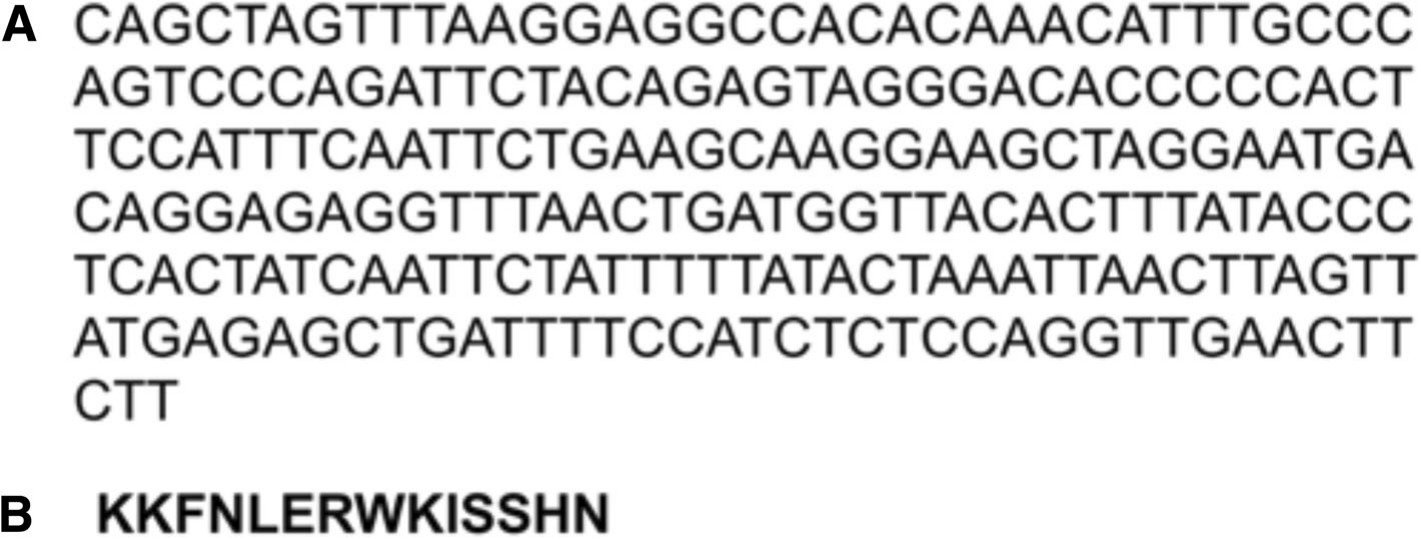
MNRR1, an autoantigen identified in BC serum. **a** The cloned 220 base pair cDNA sequence identical to that of MNRR1 (GenBank Accession #2095989 Seq1 MH071716). **b** Corresponding translated nucleotide sequence

Immunoscreening of the T7 cDNA display library of proteins identified a 220-bp cDNA clone encoding a sequence identical to MNRR1. The results of the homology search for this cDNA sequence are shown (Fig. 1a; GenBank Accession #2095989 Seq1 MH071716). BLASTn analysis resulted in the identification of expression sequence tags reflecting mRNA transcript variants 1 and 2 of *Homo sapiens* MNRR1 (also known as CHCHD2), with sequence identity of 99% and an alignment score of 438 (E value e^− 120^). BLASTx search of this transcript resulted in the identification of *H. sapiens* MNRR1 precursor isoform 1 (Fig. 1b), with associated sequence identity of 100% (E value 0.03). This clone was highly associated with the diagnosis of IDC of the breast by autoantigen microarray analysis (*P* < 0.0001).

### MNRR1 levels are higher in malignant human breast cancer tissues and derived cell lines

**Fig. 2:**
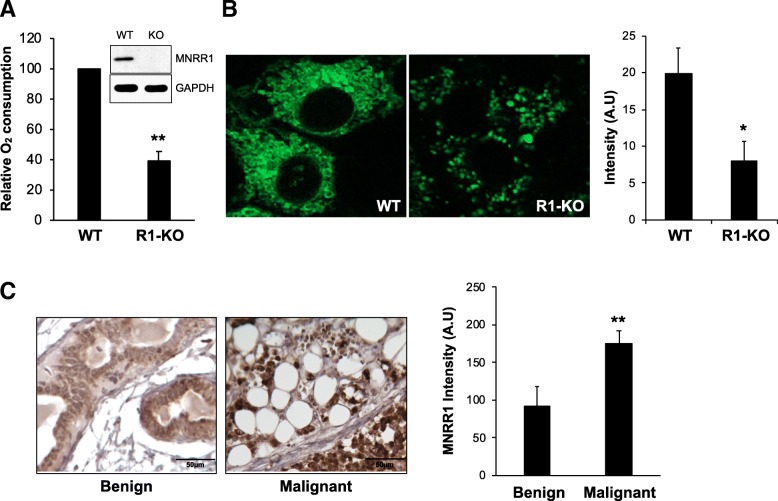
Phenotype of MNRR1 knockout cells and histochemistry. **a** Intact cellular oxygen consumption in HEK293 MNRR1-knockout (R1-KO) cells compared to controls harboring Wild Type (WT) MNRR1. **, *p* < 0.005. Inset shows an immunoblot confirming R1-KO phenotype. **b** Live staining of 293 WT and R1-KO cellular mitochondria using MitoTracker Green. Intensity of staining was calculated using ImageJ and has been shown in arbitrary units (A.U.). *, *p* < 0.05. **c** Immunohistochemistry of human invasive (IDC) (*n* = 4) and benign breast tissue (*n* = 3) stained with MNRR1. ImageJ quantitation of the staining intensity, described in Methods, is also shown. **, p < 0.005

**Fig. 3:**
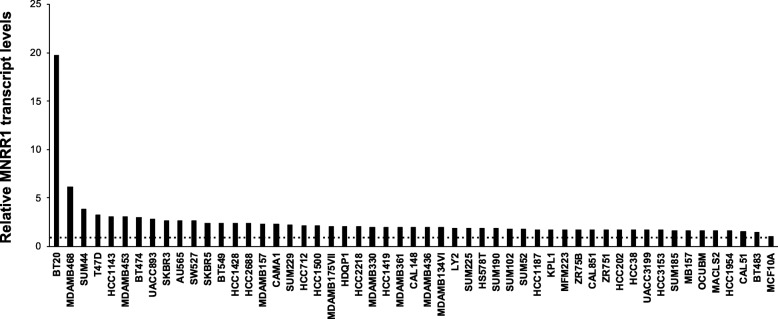
MNRR1 transcript levels in cell culture models of BC. MNRR1 transcript levels in 53 breast cancer cell lines. Values are depicted relative to the control MCF10A cell line (rightmost position), whose value is set to 1 and marked by a dotted line

Although we had previously shown the effects of MNRR1 as a regulator of mitochondrial metabolism using a short hairpin RNA (shRNA) knockdown model [8], in this work we have further examined its regulatory effects on mitochondrial function in the indicated cell lines with a knockout of this gene, R1-KO. We measured intact cellular oxygen consumption and found that it was significantly lower in the R1-KO cells compared to controls (Fig. 2a). Live staining of the WT and R1-KO cellular mitochondria using MitoTracker Green also displayed a reduction in fluorescence levels, with a loss of the mitochondrial network in the R1-KO cells, which displayed a punctate phenotype (Fig. 2b). To determine whether levels of this regulator of cellular mitochondrial function are associated with BC, we compared invasive BC versus benign breast tissue from human subjects. Upon quantitation of immunohistochemistry (IHC) staining of MNRR1, we found the levels of MNRR1 to be higher in the samples from invasive BC compared to the ones from benign breast tissue (Fig. 2c). Three benign and 4 aggressive samples were analyzed for this study with three random fields chosen for intensity quantitation for each sample. We then ascertained whether the higher MNRR1 protein levels resulted from higher transcription rates by examining the levels of MNRR1 transcripts in 53 BC cell lines and comparing them to the widely used MCF10A control [19, 20]. We found that the BC cell lines displayed a significant increase in MNRR1 transcripts ranging from 1.5 to 18-fold (Fig. 3).

### Migration of breast cancer derived cells is dependent on MNRR1 levels

**Fig. 4:**
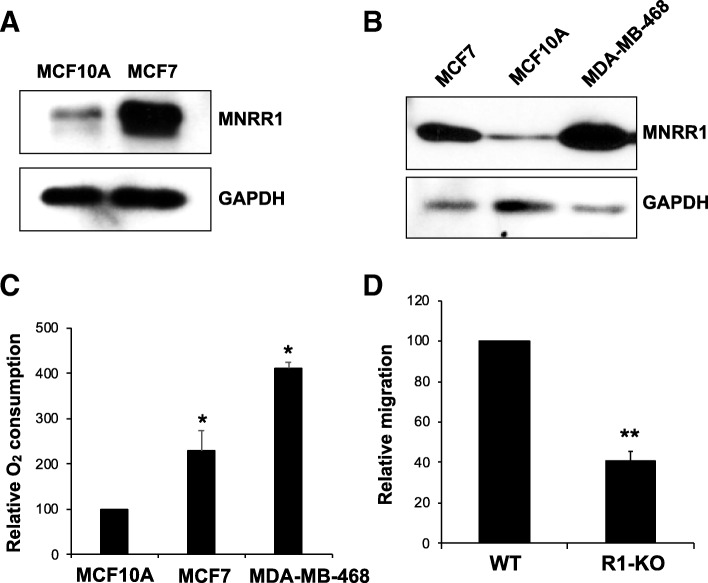
MNRR1 levels and mitochondrial function in MCF7 cells. **a** Representative immunoblot depicting MNRR1 protein levels in MCF7 and MCF10A cells. GAPDH is a loading control. **b** Comparison of MNRR1 levels among MCF10A, MCF7, and MDA-MB-468 cells. **c** Relative intact cellular oxygen consumption in MCF10A, MCF7, and MDA-MB-468 cells was measured on a Seahorse Bioanalyzer. *, p < 0.05. **d** Cell migration of WT and R1-KO MCF7 cells analyzed in a Matrigel assay as detailed in Materials and Methods. **, p < 0.005

To determine whether MNRR1 is critical for the mitochondrial phenotype in BC cells, we compared the protein levels of MNRR1 in the MCF7 BC cell line and the MDA-MB-468 line to those of control MCF10A cells. We observed that the levels of MNRR1 were higher in MCF7 cells (Fig. 4a). Moreover, MNRR1 levels were highest in MDA-MB-468 cells, followed by MCF7 and lowest in control MCF10A (Fig. 4b). In agreement with our findings on the mitochondrial function proposed for MNRR1 [7, 8], the MDA-MB-468 cells displayed the highest oxygen consumption, followed by MCF7 cells, relative to MCF10A cells (Fig. 4c). Since a key feature of IDC of the breast is its high predilection for developing metastasis to distant organs, we examined whether the higher MNRR1 levels in the invasive BC samples are required for cell migration and metastatic potential. To do so, we generated an MCF7 MNRR1-knockout cell line (R1-KOm). These knockout cells displayed a ~ 50% reduction in their ability to pass through Matrigel (Fig. 4d).

### MNRR1 is upstream of multiple genes associated with cancer metastasis

MNRR1 stands out among other genes putatively involved in breast carcinogenesis because it is a nDNA-encoded protein with important mitochondrial functions that is also upstream of other mitochondrial gene products identified by biopanning a BC cDNA library with BC sera (Table 2). All the genes listed in Table 2 contain a promoter DNA sequence similar to the sequence we have previously shown to be bound by MNRR1 [8–10].

**Table 2:** Nuclear DNA-encoded gene products recognized by the immune system as autoantigens

Antigen	Accession	MNRR1-binding sequence TTCCCACG
COA4	NM_016565.2 NP_057649.2	C**TCCCAC**C
COX7A2	NM_001865.3 NP_001856.2	A**TCCCAC**T
GAPDH	NG_007073.2	G**TCCCA**GA
GSTP1	NM_000852.3	A**TCCCA**GT
HAGH	NG_023249.1	C**TCCCA**GA
HIGD1A	NM_001099668.1 NP_001093138.1	C**TCCCAC**T
MAPK3	NG_029936.1	C**TCCCAC**C
MFF	NG_033153.1	**TTCCCA**TA
TIMM8	NG_011734.1	C**TCCCAC**A
PHB2	NM_001144831.1	A**TCCCA**AC
PKM	NG_052978.1	G**TCCCAC**C
DBI-rel protein	NM_001079862.2 NP_001073331.1	G**TCCCACG**
PRAX-1	NC_000017.11	C**TCCCAC**C
Src/SGEF	NM_015595.3	**TTCCCA**G**G**
PKC substrate	NM_000016.1 NM_002356	ND
MT-RNR Humanin	ENSG00000210082 Q8VG9	ND

Nuclear encoded genes localized to the mitochondria identified as autoantigens. Most of these genes harbor the conserved core of the MNRR1 DNA-binding sequence. Conserved residues have been shown in bold

**Fig. 5:**
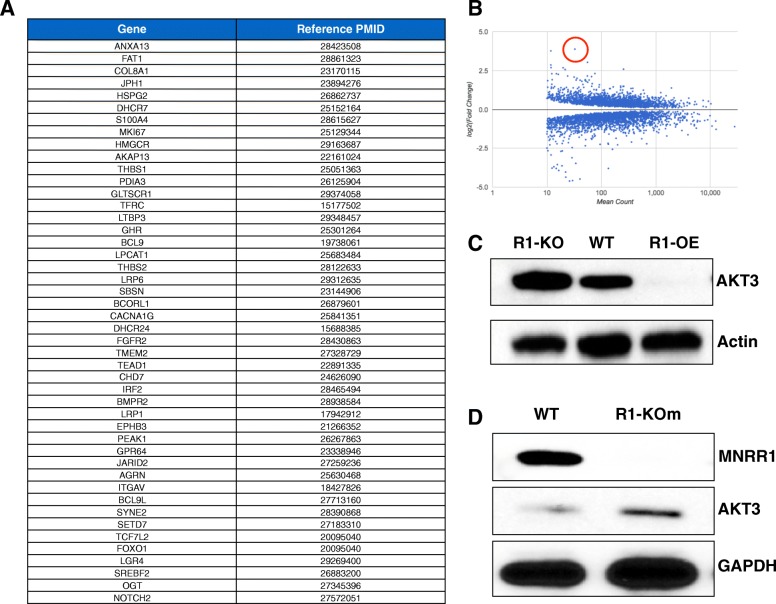
MNRR1 upstream of known metastasis-associated genes. **a** List of genes identified by RNA-Seq to be significantly downregulated in the HEK293 R1-KO cells and rescued in the R1-KO cells expressing the transcriptionally active version of MNRR1 (R1-TA). The PubMed IDs (PMID) of the studies published indicating the role of each of the genes in cancer metastasis is shown. **b** AKT3 (circled) is the most upregulated gene in the MNRR1-knockout cells. **c** Total AKT3 protein levels in WT, R1-KO, and R1-OE cells with Actin used as a loading control. **d** MCF7 cells (WT) with a knockout of MNRR1 (MCF7 R1-KOm; KO) also display an increase in total AKT3 protein levels. GAPDH is used as a loading control

MNRR1 functions in the nucleus as a transcription factor for genes harboring the conserved 13-bp DNA promoter element by binding its 8-bp core sequence along with RBPJκ [8, 9]. To identify the genes under direct control of MNRR1 that could be relevant in cancer invasiveness and metastasis, we compared RNA-Seq data obtained from HEK cells with an R1-KO versus WT HEK cells. In a second analysis, differentially regulated genes in the R1-KO cells were compared to the R1-KO cells into which a transcriptionally enhanced version of MNRR1 (MNRR1-TA) was introduced. Analyzing these two sets, we could identify 46 genes that have been previously identified as part of the cancer invasive phenotype and metastasis. Figure 5a lists these genes and the PubMed ID (PMID) for studies characterizing the importance of each gene in cancer cell metastasis. AKT3 was identified as the most upregulated gene in the R1-KO cells (Fig. 5b). Furthermore, the level of AKT3 in these cells is inversely proportional to the aggressive metastatic phenotype in BC cells [21, 22]. We thus determined the level of AKT3 in cells with either a WT, R1-KO, or R1-overexpressed (R1-OE) phenotype and found that AKT3 protein levels were inversely proportional to the R1 genotype (Fig. 5c), supporting the RNA-Seq data. Importantly, we asked whether this were also true in the BC model using the MCF7 R1-KOm cells. AKT3 levels were found to be higher in the knockout cells (Fig. 5d).

### MNRR1 affects Rictor and PGC-1α levels in breast cancer cells

**Fig. 6:**
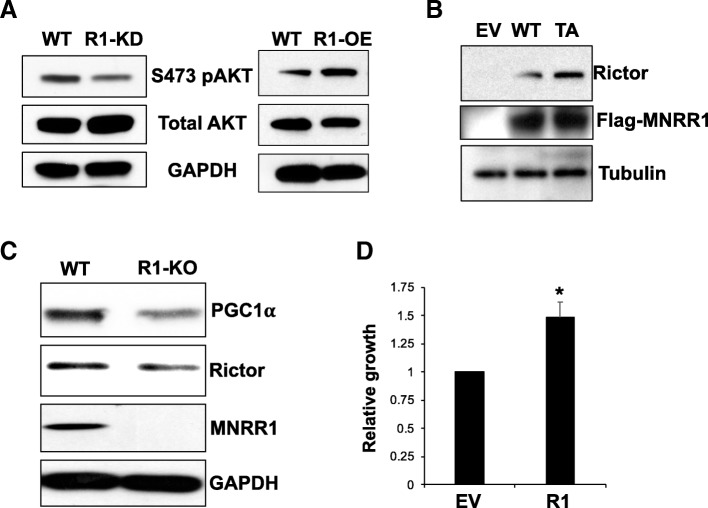
MNRR1 and cellular growth. **a** S473 pAKT levels in MNRR1-knockdown (R1-KD) and MNRR1-OE (R1-OE) cells compared to WT controls. Total AKT levels do not change. GAPDH is used as a loading control. **b** Total Rictor protein levels in WT cells expressing either an empty vector (EV), Flag-tagged WT-MNRR1 (WT), or flag tagged transcriptionally active MNRR1 (TA). Tubulin is used as a loading control. Flag levels indicate transfection efficiency between WT and TA MNRR1. **c** PGC-1α and Rictor protein levels in MCF7 cells with a knockout of MNRR1. GAPDH is used as a loading control. **d** Cell growth between MCF10A cells transfected with either an empty vector (EV) or MNRR1 (R1) expression plasmid. The Cell Titre96 assay was measured spectrophotometrically. *p* = 0.03

Our initial characterization of MNRR1 identified a slow growth phenotype in MNRR1-shRNA knockdown (MNRR1-KD) cells [8]. Since the AKT signaling pathway is known to be responsible for cell proliferation and migration [23, 24], and Rictor has been identified as the kinase that phosphorylates and activates AKT at S473 [25], we explored the participation of MNRR1 in this process. We found that the promoter for Rictor harbors the conserved oxygen response element to which MNRR1 binds, suggesting that Rictor is a direct transcriptional target for MNRR1. We found that R1-KO cells are defective in their levels of activated pS473-AKT. In addition, cells overexpressing MNRR1 (R1-OE) show increased pAKT levels (Fig. 6a). To investigate whether MNRR1 affects levels of Rictor, we transfected HEK cells with either an empty vector (EV), WT-MNRR1 R1, or MNRR1-TA. We found that Rictor protein levels increase in cells expressing WT-MNRR1 and increase even further in cells expressing MNRR1-TA (Fig. 6b). We also examined the levels of Rictor in WT and MCF7-R1-KOm cells and found lower levels of Rictor in the MCF7-R1-KOm cells (Fig. 6c). We next focused on PGC-1α, a key protein responsible for mitochondrial biogenesis [26], since recent studies have highlighted the role of this protein in BC metastasis [27]. For example, metastasizing cells undergo a switch in their metabolism towards a predominantly oxidative mode via mitochondrial biogenesis mediated by increased PGC-1α levels [27, 28]. We tested the R1-KOm cells for the levels of PGC-1α and found it to be reduced (by ~ 50%) in the R1-KOm cells (Fig. 6c). We then examined whether MNRR1 affects growth rate by comparing the growth of control MCF10A cells after introducing a vector expressing MNRR1; by 48 h post transfection MCF10A cells expressing MNRR1 displayed significantly faster growth than control cells with an empty vector (Fig. 6d). The growth enhancing effect of MNRR1 expression was also observed in HEK cells (data not shown).

## Discussion

The identification of MNRR1 cDNA in our phage BC library, the association of this phage with the diagnosis of IDC of the breast in the autoantigen microarray, the increased expression of this mitochondrial protein in histologically confirmed malignant breast tissue, and the correlation of MNRR1 expression with markers of metastatic BC, all suggest that this protein is important in the pathophysiology of BC and, in particular, its metastasis. For example, the levels of AKT3, a negative regulator of BC metastasis [21, 22], were found to be inversely proportional to MNRR1 levels. Furthermore, RNA-Seq data in HEK cells and proteomic data in BC cell lines, taken together, are also consistent with the role of MNRR1 as a key regulator of genes involved in an aggressive phenotype.

The participation of a regulator of mitochondrial respiration such as MNRR1 in breast metastatic disease is in agreement with previous work identifying an oxidative phenotype in metastatic BC cells [27, 29]. At least two specific features of mitochondria besides their efficient production of ATP have emerged. One is the need for a controlled but elevated level of reactive oxygen species (ROS) production, in part arising at mitochondrial complexes I and III [30]. We have reported that several subunits of complex I are targeted by AMAs [6]. The ROS produced are needed for cellular signaling for both growth and metastasis, for instance by increasing SRC/PYK2 protein kinase signaling [28]. We have noted that several subunits of complex I of the ETC are targeted by autoantibodies (Table 1). In addition, we have found autoreactivity to several nDNA-encoded mitochondrial proteins including PHB2 and HIGD1A, which have been implicated in the pathogenesis of BC, as well the exchange factor SGEF, which suggests abnormal Src signaling in breast carcinogenesis (Table 2; unpublished). An important conclusion is that MNRR1, in addition to being an activator of mitochondrial function, also promotes cell migration [31], suggesting that both its mitochondrial and its nuclear functions are involved in the development of BC metastasis.

Our previous work and the data on PGC-1α (Fig. 6c) show that MNRR1 promotes mitochondrial respiration [7, 8]. In addition, MNRR1 promotes other features of a metastatic phenotype. These include the import of MNRR1 itself, since the presence of autoantibodies to TIMM8 in BC sera (Table 2) suggests the possibility of a disturbance in protein import, the inhibition of apoptosis through the interaction of MNRR1 with Bcl-xL [32], preventing outer mitochondrial membrane permeabilization, and the promotion of cellular migration [31].

*MNRR1* expression has also been linked to lung cancer [11] and to follicular carcinoma of the thyroid [33]. In addition, studies on the pathogenesis of hepatocellular carcinoma have suggested a role for the involvement of MNRR1 in the process of carcinogenesis, mediated through the interaction of cyclic adenosine monophosphate response element-binding protein (CREB) with the *MNRR1* promoter, leading to an increased level of its expression [12]. In agreement with our proposal, proteomic analysis of mammosphere models of BC has demonstrated a high degree of MNRR1 upregulation in this system [34]. These findings suggest both the involvement of MNRR1 in breast carcinogenesis and a potential role as a biomarker of aggressive BC. In this work we report that autoreactivity to MNRR1 is highly associated with the diagnosis of IDC of the breast by autoantigen microarray analysis. We further show here that properties associated with carcinogenesis are directly regulated by MNRR1.

We have previously reported the detection of a distinctive anti-mitochondria antibody profile in invasive BC, but also in DCIS of the breast and in a small group of healthy women with suspicious mammography findings but without detected BC on biopsy, importantly suggesting that autoantibodies are formed during the pre-malignant stage [5]. Furthermore, we have reported AMAs targeting mtDNA-encoded components of the ETC, which may represent an expression of mitochondrial autoimmunity [6]. It is likely that MNRR1 autoreactivity, as well as the autoreactivity observed in other nDNA-encoded mitochondrial proteins as well as the AMAs targeting the components of the ETC ([6] and Table 1), are expressions of mitochondrial autoimmunity in BC. We have now shown that IHC using a specific antibody to MNRR1 can contribute to distinguish malignant from benign breast tissue. This is important because mammographic assessment of women undergoing annual screening mammography is performed using the Breast Imaging and Reporting Data System (BI-RADS) [35] wherein the standard of care for a suspicious mammography assessment (BI-RADS4) is to follow with breast biopsy [36]. Only about 20% of these women are found to have BC whereas in about 80% the pathological exam reveals BBD. These healthy women are usually reassured and recommended to continue annual or bi-annual routine mammography study. We suggest that autoantibodies to a panel of mitochondrial antibodies including MNRR1, together with positive IHC staining for this protein, and perhaps for other nDNA- and mtDNA-encoded mitochondrial antigens, on breast biopsies performed routinely on women having a BIRADS4 assessment might indicate that the subject is at a higher risk for BC than is suggested by a BBD diagnosis found at breast biopsy. Since our study indicates that MNRR1 participates in breast carcinogenesis, our findings strongly suggest the need for further studies to elucidate the contribution of MNRR1 and other nDNA- and mtDNA-encoded mitochondrial antigens to the diagnosis of early BC.

In the context of the spectrum of autoantibodies reported in BC sera [4–6], autoreactivity to MNRR1 and other proteins targeted by autoantibodies have potential as early markers of BC metastasis. We propose that a panel of mtDNA- and nDNA-encoded mitochondrial antigens should be evaluated for utility in BC screening to increase early BC detection and perhaps to identify healthy women at higher risk for BC. Based on the studies on mitochondrial function in BC and the findings reported here, aggressive cancer cells shift to an oxidative metabolism during a metastatic change [28, 37]. An important feature in breast carcinogenesis is the need for TCA cycle-derived intermediates for synthesis of growth precursors. To this effect, some of the AMAs we found in BC sera target key enzymes involved in carbohydrate metabolism in the mitochondria such as GAPDH and GSTP1 (Table 2). Detection of AMAs in BC indicates an immune response triggered by the host against antigens developing in the cancerous cells. There is an established causal relationship between chronic inflammation and a number of solid tumors [38, 39]. In other solid tumors such as BC chronic inflammatory changes are often found although in the absence of a diagnosable inflammatory or infectious event. Since auto-immune damage is known to produce significant cellular damage and chronic inflammation in the RADs [40, 41], we have proposed that autoimmune damage to the breast and perhaps as well to other solid tumors, induces an inflammatory milieu likely to promote carcinogenesis via multiple regulatory functions in the mitochondria such as oxidative phosphorylation, ROS signaling, and one-carbon metabolism [6].

AKT is a signaling protein that has been shown to play a key role in cancer progression and metastasis [42]. Akt has three isoforms, AKT1, AKT2, and AKT3, each with a distinct function in cell physiology. Of the three isoforms, AKT3 was initially proposed to be involved in CNS development [43]. Newer studies have shown it to have an inhibitory effect on cancer growth [44]. In breast cancer cells, downregulation of AKT3 has also been shown to enhance metastasis [21, 22]. AKT3 was the most upregulated gene in the MNRR1-KO cells and may be one potential pathway for our observation of reduced cell migration. In addition, it also highlights the importance of identifying selective AKT inhibitors as a therapeutic in BC.

## Conclusions

MNRR1 regulates multiple genes that function in cell migration required for cancer metastasis and is higher in cell lines derived from tumors previously shown to have enhanced metastatic potential. Since MNRR1 was identified as an autoantigen in breast carcinogenesis, and the other mitochondrial autoantigens are potentially downstream of MNRR1, the present data strongly support the role of MNRR1 and mitochondrial autoimmunity in breast carcinogenesis.

In this work we focus on both the direct participation of MNRR1 in breast carcinogenesis and the demonstration of autoreactivity to this protein in BC sera and its expression in BC tissue as an example of mitochondrial autoimmunity in BC. However, it is clear that MNRR1 is not the only mitochondrial protein involved in breast carcinogenesis since we found autoreactivity to a number of mtDNA- and nDNA-encoded mitochondrial proteins in BC sera (Tables 1 and 2) that suggest the widespread autoreactivity to mitochondrial antigens is the expression of autoimmunity in BC. Finally, the identification of MNRR1 in both cell lines and patient samples of aggressive BC suggests that it could serve as both a biomarker and a new drug target.

## Abbreviations

AMAs: Anti-mitochondrial antibodies
BBD: Benign breast disease
BC: Breast cancer
BI-RADS: Breast Imaging and Reporting Data System
DCIS: Ductal carcinoma in situ
ETC: Electron transport chain
IDC: Invasive ductal carcinoma
MCF7-R1-KOm: MNRR1 knockout in MCF7 cells
MNRR1: Mitochondrial-Nuclear Retrograde Regulator 1 (also called CHCHD2)
MNRR1-KD: MNRR1-shRNA knockdown
MNRR1-KO: CRISPR knockout of MNRR1
MNRR1-TA: Transcriptionally active version of MNRR1
mtDNA: Mitochondrial DNA
nDNA: Nuclear DNA
R1-OE: Overexpressed MNRR1
RIN: RNA Integrity Number
WT-MNRR1: Wild-type MNRR1
